# A laboratory system for rearing Simuliidae (Diptera) under simulated lotic environments

**DOI:** 10.7717/peerj.21193

**Published:** 2026-06-08

**Authors:** Ruan Guimarães, Wilian Rodrigues da Costa Marinho, Arieli Bernardo Portugal, Rodrigo Nunes-da-Fonseca

**Affiliations:** NUPEM-UFRJ-Macaé, Universidade Federal do Rio de Janeiro, Macaé, RJ, Brazil

**Keywords:** Blackfly, Aquatic bioterium, Behavior, Larval rearing

## Abstract

**Background:**

Black flies (Diptera: Simuliidae) are ecologically important components of lotic ecosystems and a public health concern due to their aggressive biting behavior, pathogen transmission, and induction of allergic and inflammatory reactions in humans and livestock. Despite their relevance, experimental research on Simuliidae remains limited compared with other dipteran vectors, largely because their immature stages require specific aquatic conditions that are difficult to reproduce in the laboratory. Larval development depends on continuous water flow, high dissolved oxygen, and suspended particulate food, factors that have hindered the establishment of reproducible rearing protocols. Here, we assess the feasibility and efficiency of rearing Simuliidae through their aquatic stages under controlled laboratory conditions approximating natural lotic environments.

**Methods:**

Egg masses were collected from submerged substrates in the São Pedro River (Macaé, Rio de Janeiro, Brazil) and transported to the Institute of Biodiversity and Sustainability (NUPEM/UFRJ) aquatic bioterium. Eggs were incubated in aerated containers until hatching, and larvae were transferred to a circular rearing system generating continuous unidirectional flow using submersible pumps. Physicochemical parameters were monitored daily (20–25 °C; pH 7.0; ammonia and nitrite ≤ 0.25 ppm). Larvae were fed a standardized suspension of yeast, spirulina, and chlorella. Eleven independent experiments monitored development from hatching to pupation. Additional assays tested larval densities (50, 100, 200 individuals) and survival following flow interruption. Pupal viability was assessed through adult emergence.

**Results:**

Mean egg hatching success was 74%. Under continuous flow, larval survival averaged 92%, with pupation occurring within 13–15 days. Adult emergence reached 92.5%, indicating high pupal viability. Increased density caused only modest survival reduction, demonstrating system robustness. In contrast, flow interruption caused rapid mortality after 24 h, confirming strict dependence on oxygenated flow. Morphological identification revealed five species—*Simulium pertinax*, *S. subpallidum*, *S. nigrimanum*, *S. hirtipupa*, and *S. rubritorax* —indicating frequent multispecies oviposition.

**Discussion:**

Simuliidae can be reliably reared through aquatic stages under laboratory conditions when hydrodynamic and physicochemical parameters are controlled. Although long-term colonization was not achieved, the protocol provides a reproducible framework for experimental studies on simuliid development, physiology, and biting-associated allergic responses.

## Introduction

Black flies (Diptera: Simuliidae) are aquatic insects whose immature stages develop exclusively in lotic environments characterized by continuous flow, high dissolved oxygen, and suspended particulate matter. Larvae and pupae remain attached to submerged substrates, while adults are aerial, with females of many species exhibiting aggressive hematophagous behavior. At a global scale, simuliids are widely distributed across temperate, tropical, and subpolar regions and are particularly diverse in tropical freshwater systems (Currie & Adler, 2008; Adler & McCreadie, 2019; Hamada, Pepinelli & Gil-Azevedo, 2022). In Brazil and other tropical regions, black flies represent a persistent public health concern due to intense biting pressure, severe allergic and inflammatory reactions, and their involvement in pathogen transmission or disease promotion (Viviani, Strieder & Spies, 2012; Adler, 2025).

Beyond classical biological transmission, simuliids have long been considered candidates for the “promoter arthropod” hypothesis, whereby frequent and traumatic biting facilitates pathogen transmission without obligate development within the vector. This concept has recently regained attention following epidemiological analyses linking simuliid abundance to the co-occurrence of *Mansonella ozzardi* infections and hepatitis B virus prevalence in the Brazilian Amazon (Araújo, Balieiro & Crainey, 2024). Together with the capacity of simuliid salivary secretions to induce hypersensitivity reactions and autoimmune-like dermatological syndromes, these findings reinforce the need for experimental systems that allow controlled investigation of simuliid biology, physiology, and vector–host interactions (Criado et al., 2025).

The phylogenetic position of black flies (Diptera: Simuliidae) within the infraorder Culicomorpha, a major lineage of the suborder Nematocera, places them in close evolutionary proximity to mosquitoes and other hematophagous Diptera (Ribeiro, Mans & Arcá, 2010; Kutty et al., 2018). This position establishes Simuliidae as a critical comparative outgroup for studies addressing the evolution of blood feeding, vectorial capacity, and host–pathogen interactions across dipteran lineages. Phylogenomic analyses resolving deep relationships within Culicomorpha have reinforced the value of black flies for comparative evolutionary frameworks aimed at understanding the independent origins and diversification of hematophagy and associated salivary, behavioral, and physiological traits (Ribeiro, Mans & Arcá, 2010). Closing existing gaps in our biological and experimental knowledge of Simuliidae therefore has the potential to substantially advance both evolutionary biology and medical entomology, with direct implications for vector management strategies. Despite their relevance as disease vectors, economic nuisances, and integral components of freshwater ecosystems, experimental studies focusing on simuliid embryology, biochemistry, and physiological responses to environmental stress remain scarce or entirely absent when compared with other dipteran vectors.

Despite their ecological, evolutionary and medical relevance, Simuliidae remain markedly underrepresented in laboratory-based research when compared with other dipteran vectors such as mosquitoes and sand flies. Stable laboratory colonies exist for many medically important Diptera, supporting experimental studies on development, physiology, toxicology, and pathogen interactions. In contrast, long-term laboratory colonization of black flies has proven exceptionally difficult, even for major vector species, despite sustained efforts by international public-health programs (Edman & Simmons, 1985; Gray & Noblet, 2014). This limitation has restricted experimental access to early developmental stages and hindered the integration of simuliids into comparative vector biology.

The primary bottleneck lies in the larval stage. Simuliid larvae are obligate filter feeders that rely on continuous, directional water flow to deliver suspended organic particles and maintain adequate oxygenation. They are highly sensitive to physicochemical instability, particularly ammonia and nitrite accumulation, and display stage-specific microhabitat preferences associated with increasing flow velocities as development proceeds (Brenner & Cupp, 1980; Buffolo et al., 2016; Figueiro et al., 2021). Conventional laboratory aquaria and filtration systems are poorly suited to meet these combined demands, as they tend to remove suspended food particles and fail to reproduce stable hydrodynamic regimes typical of natural streams (Gray & Noblet, 2014; Martins et al., 2017).

Previous attempts to rear simuliids under laboratory conditions have employed bubble curtains, inclined channels, and closed circulation systems, often achieving partial development but with limited reproducibility and high mortality at later stages (Edman & Simmons, 1985; Gray & Noblet, 2014). Quantitative assessments of survival, pupation, and adult emergence across replicated experiments remain scarce, particularly for neotropical species, limiting the use of simuliids as experimental organisms (Srisuka et al., 2015; Hamada, Pepinelli & Gil-Azevedo, 2022).

Here, we evaluate a controlled laboratory rearing system designed to approximate key features of natural lotic habitats, with emphasis on continuous water flow, physicochemical stability, and sustained availability of suspended food particles. Rather than attempting long-term colonization, our objective was to assess the feasibility, reliability, and constraints of short-term rearing from egg hatching to adult emergence across multiple independent trials. By quantifying hatching success, larval survival, pupation, and adult eclosion, and by documenting multispecies emergence from natural egg masses, we provide a reproducible methodological framework for experimental studies on Simuliidae.

**Figure 1:**
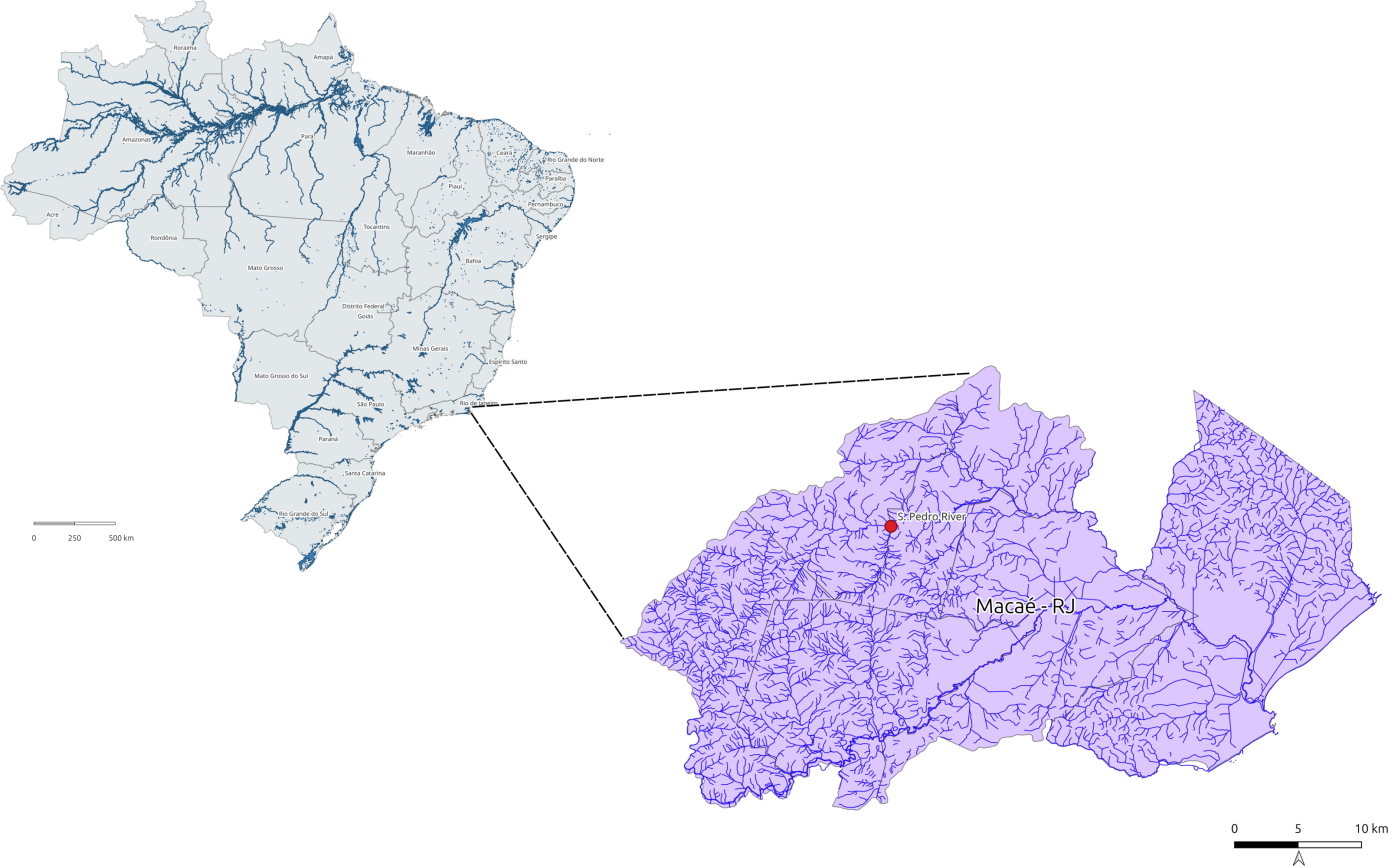
Egg collection site of *Simulium* species. Geographical location of the São Pedro River (22°14′18″S, 42°03′27″W), the main tributary of the Macaé River in southeastern Brazil, where black fly egg masses were collected from submerged rocky substrates. The inset shows the regional hydrographic network within the rural area of Macaé, Rio de Janeiro. The sampling point is marked in red.

## Materials & Methods

### Study area and egg collection

**Figure 2:**
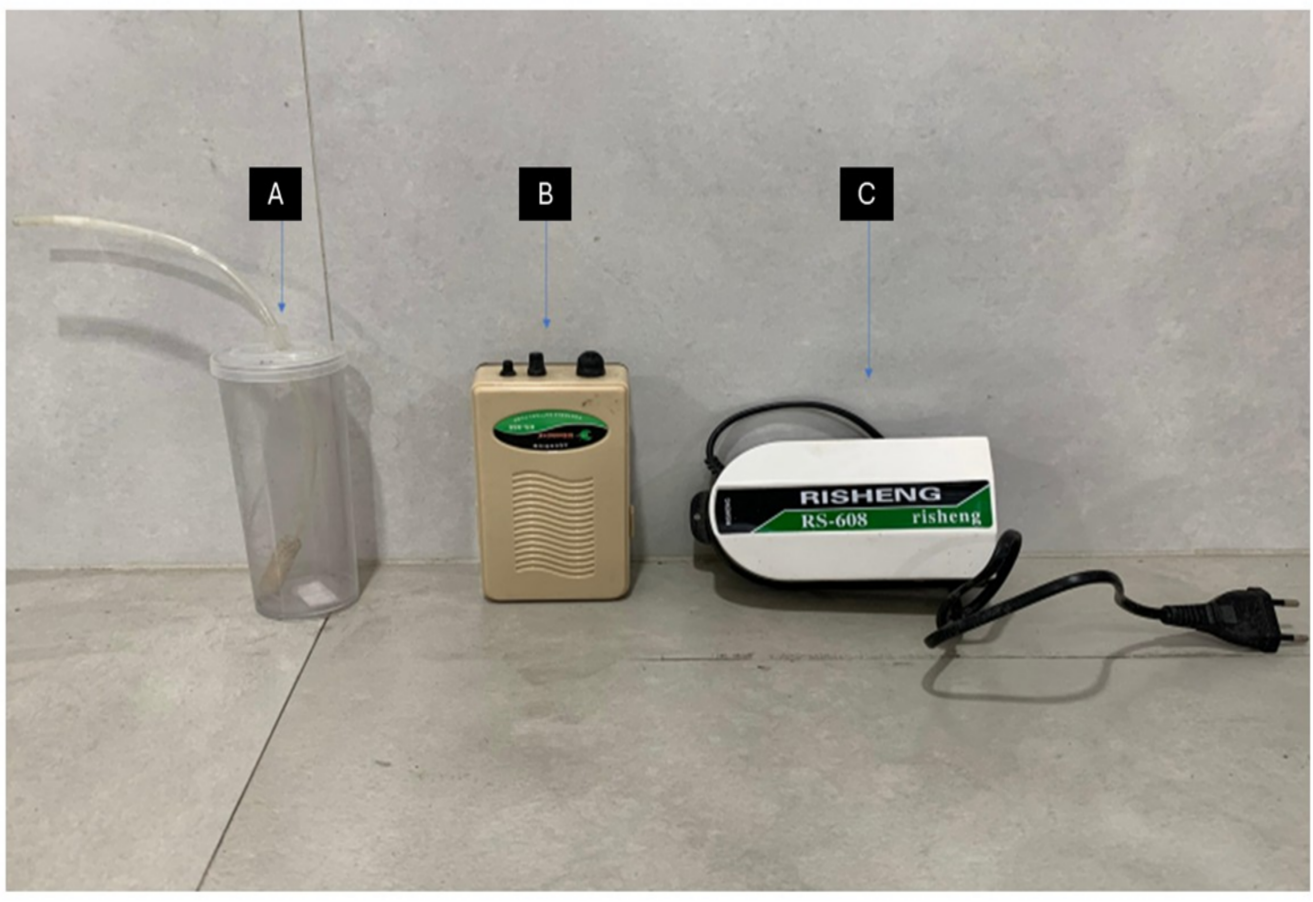
Egg transport container. (A) Portable cylindrical container (700 mL) used for egg collection and transport. (B) BOYU D-200 portable aerator (2 L min^−1^) ensuring oxygenation during transport. (C) Virgo AR60 fixed aeration pump used in the hatching aquarium.

Egg masses of Simuliidae were collected from egg masses adhered to rocky substrates in fast-flowing sections of the São Pedro River, municipality of Macaé, Rio de Janeiro State, Brazil (22°14′18″S, 42°03′27″W; Fig. 1). Collections were performed in shallow lotic areas where simuliid oviposition is commonly observed, consistent with previous descriptions of oviposition behavior in neotropical black flies (Hamada, Adler & McCreadie, 2018; Adler & McCreadie, 2019). Egg masses were carefully removed using metal tweezers to avoid mechanical damage and immediately transferred to a cylindrical plastic container (Fig. 2A) containing 700 mL of water collected at the same site. During transport, the container was continuously aerated using a portable oxygenation pump (BOYU D-200; flow rate 2 L min^−1^) (Fig. 2B). Eggs were maintained under these conditions for up to six hours during transport to the Aquatic Organisms Bioterium (BOA) of the Institute of Biodiversity and Sustainability (NUPEM/UFRJ). Upon arrival, egg masses were gently separated, and eggs were transferred to a dedicated hatching aquarium, for further incubation and maturation with an aeration connector attached to an air pump (Virgo AR60) (Fig. 2C),

### Egg hatching assessment

**Figure 3:**
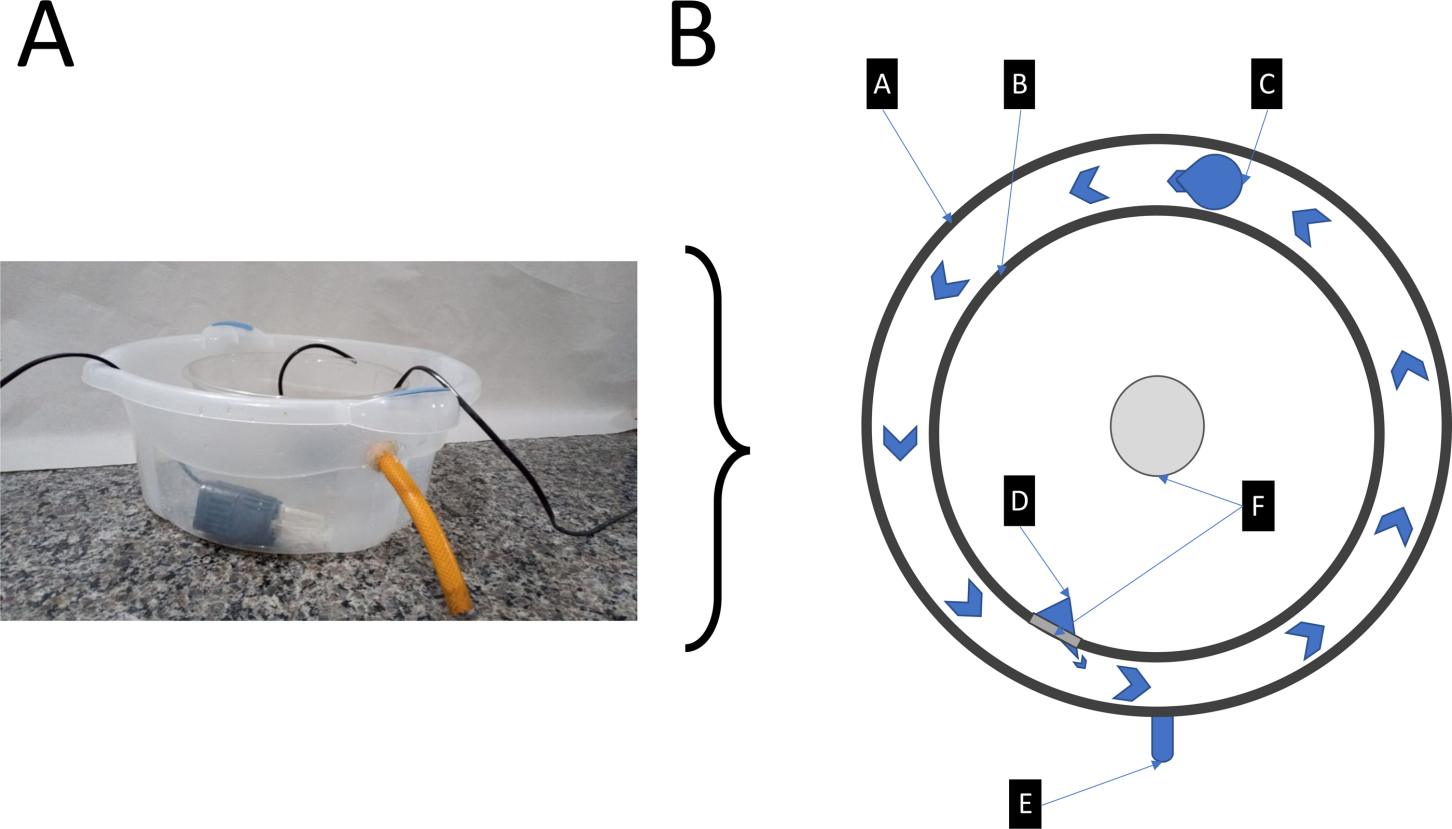
Dual-container circular water-flow system for laboratory rearing of *Simulium* larvae. (A) Photograph of the experimental larval rearing apparatus composed of two concentric containers installed inside a larger aquarium reservoir. Water circulation is generated using a high-flow submersible pump, while continuous aeration maintains oxygenation and prevents stagnation. (B) Schematic representation of the system illustrating its hydrodynamic organization. The outer container (A) functions as the main water reservoir, whereas the inner container (B) defines the larval rearing chamber. Water movement is driven by a submersible circulation pump (C; SB1000A, 1,500 L h^1^), producing a continuous circular flow that mimics natural stream conditions required for *Simulium* larval attachment and feeding. Additional aeration is provided by a submerged oxygenation pump (D; 3,000 L h^1^). Excess water exits through an overflow drainage pipe (E), enabling partial water renewal. Perforations in the inner container floor (F) allow controlled water exchange between compartments while maintaining directional flow and preventing larval accumulation in low-oxygen zones. The system generates a peripheral high-flow region and a central low-flow zone, promoting stable circulation and efficient waste removal.

Egg hatching was monitored to evaluate the efficiency and reproducibility of the incubation setup. For each hatching assay, 100 eggs from a recently laid pustule were gently separated and counted under a binocular stereomicroscope (ECZ-BLACK-BI-45-BI) using an 8-key manual blood cell counter, a technique commonly used for small arthropod egg enumeration (Ward, 1992). Eggs were then transferred to a dedicated cylindrical plastic hatching container (25 cm height × 8 cm diameter) containing 500 mL of river water collected at the sampling site (Figs. 3A, 3B). The container was equipped with a localized bubble curtain and mild water current within the vessel. Eggs were kept adjacent to this bubble curtain to maintain oxygenation and suspension of fine particulate matter, conditions that have been shown to promote hatching and early larval activity in lotic Diptera (Brenner & Cupp, 1980; Edman & Simmons, 1985). The use of directed aeration to approximate natural flow regimes follows approaches previously reported for experimental rearing of simuliid and other lotic larvae under controlled conditions.

Eggs were incubated for seven days at the Aquatic Organisms Bioterium (BOA) under controlled conditions. Water quality parameters were monitored daily throughout the incubation period: temperature was maintained at 20 °C, pH remained near neutrality (≈ 7), and ammonia and nitrite concentrations did not exceed 0.25 ppm. Regular monitoring of these parameters is essential, as elevated nitrogenous waste products and pH shifts are known to impair egg development and larval viability in aquatic dipterans (Buffolo et al., 2016; Hamada, Adler & McCreadie, 2018).

At the end of the incubation period, hatched larvae were immediately transferred to 70% ethanol for fixation and subsequent counting, a standard approach to quantify hatching success in experimental entomology without compromising live-rearing experiments (Gray & Noblet, 2014). Larvae preserved in ethanol were used exclusively for hatching-rate quantification and were not introduced into larval rearing systems. Independent egg incubation assays were performed to supply live larvae for all subsequent larval rearing trials.

### Larval rearing and filtration system

Larval rearing was performed in a custom-designed aquarium system intended to reproduce key hydrodynamic and physicochemical features of natural lotic habitats. Fifty newly hatched larvae were transferred to a circular rearing unit composed of two concentric polyethylene containers. The inner container measured 25 cm in diameter and 17 cm in height, while the outer container measured 35 cm in diameter and 17 cm in height, with a lateral water outlet positioned 10 cm from the base. The system was filled with 8 L of river water collected at the sampling site.

Directional flow was generated by two submersible pumps (JVP-120 and SB1000A) positioned between the container walls, operating at nominal flow rates of 3,000 L h^−1^ and 1,000 L h^−1^, respectively. Pumps were oriented clockwise to generate continuous circular flow, and one unit was coupled to an aeration system to ensure high dissolved oxygen availability (Fig. 3). Continuous directional flow is essential for simuliid larval attachment and filter-feeding behavior and represents a major limiting factor in laboratory rearing (Brenner & Cupp, 1980; Gray & Noblet, 2014; Adler & McCreadie, 2019).

A biological filtration system was implemented to maintain water quality. Filtration was carried out using a 42 L rectangular aquarium containing a cylindrical filter packed with layers of perlon and ceramic rings colonized by nitrifying bacteria. A submersible pump (S300; 580 L h^−1^) positioned above the filter provided water circulation and aeration. Stones collected from the natural habitat, containing native algae and microbial communities, were added to promote acclimatization and assist nitrate retention, following established practices for aquatic insect rearing (Gray & Noblet, 2014). An 80 L storage drum equipped with activated charcoal filtration was used for chemical filtration and water storage.

Physicochemical parameters were monitored daily. Temperature, pH, and dissolved oxygen were measured using a portable oximeter (Kr86021), while ammonia and nitrite concentrations were assessed using commercial reagent kits (Labcon). Experimental conditions were maintained at 25 °C, pH 7.0, and ammonia and nitrite concentrations ≤ 0.25 ppm, thresholds consistent with simuliid larval tolerance reported in previous studies (Buffolo et al., 2016; Hamada, Adler & McCreadie, 2018). Partial water renewal was performed daily, with effluent passed through the biological filtration system and subsequently stored in the 80 L reservoir for reuse.

### Larval feeding

Larvae were fed daily with a standardized suspension composed of 0.1 g of baker’s yeast (*Saccharomyces cerevisiae*), 0.1 g of ground spirulina, and 0.1 g of powdered *Chlorella* algae. The mixture was diluted in 15 mL of river water from the collection site prior to addition to the rearing aquarium. This feeding regime was designed to maintain suspended particulate matter in the water column, mimicking the natural filter-feeding conditions of simuliid larvae and following formulations previously employed in laboratory rearing attempts (Pegoraro, 1993; Cunha et al., 2011; Adler & McCreadie, 2019).

### Larval density assays

Larval density assays were conducted to evaluate the carrying capacity and robustness of the rearing system under increasing biological load. Four independent experiments were performed. In each experiment, larvae originating from a single egg mass were randomly distributed into three rearing aquaria at initial densities of 50, 100, and 200 larvae per unit. All treatments were maintained under identical physicochemical conditions, flow regime, and feeding schedule. Larvae were monitored daily until pupation, and survival rates were calculated as the proportion of individuals reaching the pupal stage. Density-dependent effects on simuliid larval survival and development have been previously reported in both laboratory and natural settings and were considered in the experimental design (Brenner & Cupp, 1980; Srisuka et al., 2015; Hamada, Pepinelli & Gil-Azevedo, 2022).

### Survival without aeration and water circulation

Larval dependence on aeration and water circulation was evaluated in a flow-interruption experiment. Larvae were reared under standard conditions until reaching a developmental stage close to pupation and then divided into two aquaria containing 50 larvae each. After a 30 min acclimatization period, aeration and water circulation were interrupted in one aquarium, while the control aquarium remained under continuous flow. Larval survival was assessed at 6, 12, 24, and 48 h, with dead specimens removed at each time point. This experimental design was based on the well-established reliance of simuliid larvae on flow-mediated oxygenation and food delivery (Brenner & Cupp, 1980; Adler & McCreadie, 2019).

### Pupal emergence

Pupal viability was assessed by transferring pupae produced in the rearing system to individual emergence containers. Pupae were placed in plastic containers lined with moistened cotton at the base and covered with mosquito netting to prevent escape while maintaining high humidity (Fig. 2C). Four independent trials were conducted, each with 10 pupae, maintained at 25 °C. Adult eclosion was recorded daily. This setup follows established protocols for assessing simuliid pupal emergence under laboratory conditions (Gray & Noblet, 2014).

### Species identification

Species identification was performed based on morphological characteristics of larvae and adults using standard taxonomic keys for Neotropical Simuliidae. Priority was given to late-instar larvae and emerged adults, which provide the most reliable diagnostic characters for species-level identification. Larval identification focused on traits of the head capsule, including cephalic apotome patterns, hypostomal teeth, and postgenal cleft morphology, as well as thoracic and abdominal features. When available, adults were examined for diagnostic characters of the antennae, legs, and genitalia. Taxonomic assignments followed current nomenclature and systematic treatments for the group (Adler & Crosskey, 2020; Hamada, Pepinelli & Gil-Azevedo, 2022; Adler, 2025).

### Data analysis

Larval survival was compared between aeration treatments (aeration on *vs.* off) across four time points (6, 12, 24, and 48 h), and the interaction between these factors was evaluated using a two-way analysis of variance (ANOVA). Statistical analyses were performed using GraphPad Prism (version 9). Assumptions of normality and homoscedasticity were evaluated prior to analysis, and statistical significance was assessed at a 5% probability level.

## Results

### Performance of a laboratory system for rearing *Simulium* spp. under controlled conditions

**Figure 4:**
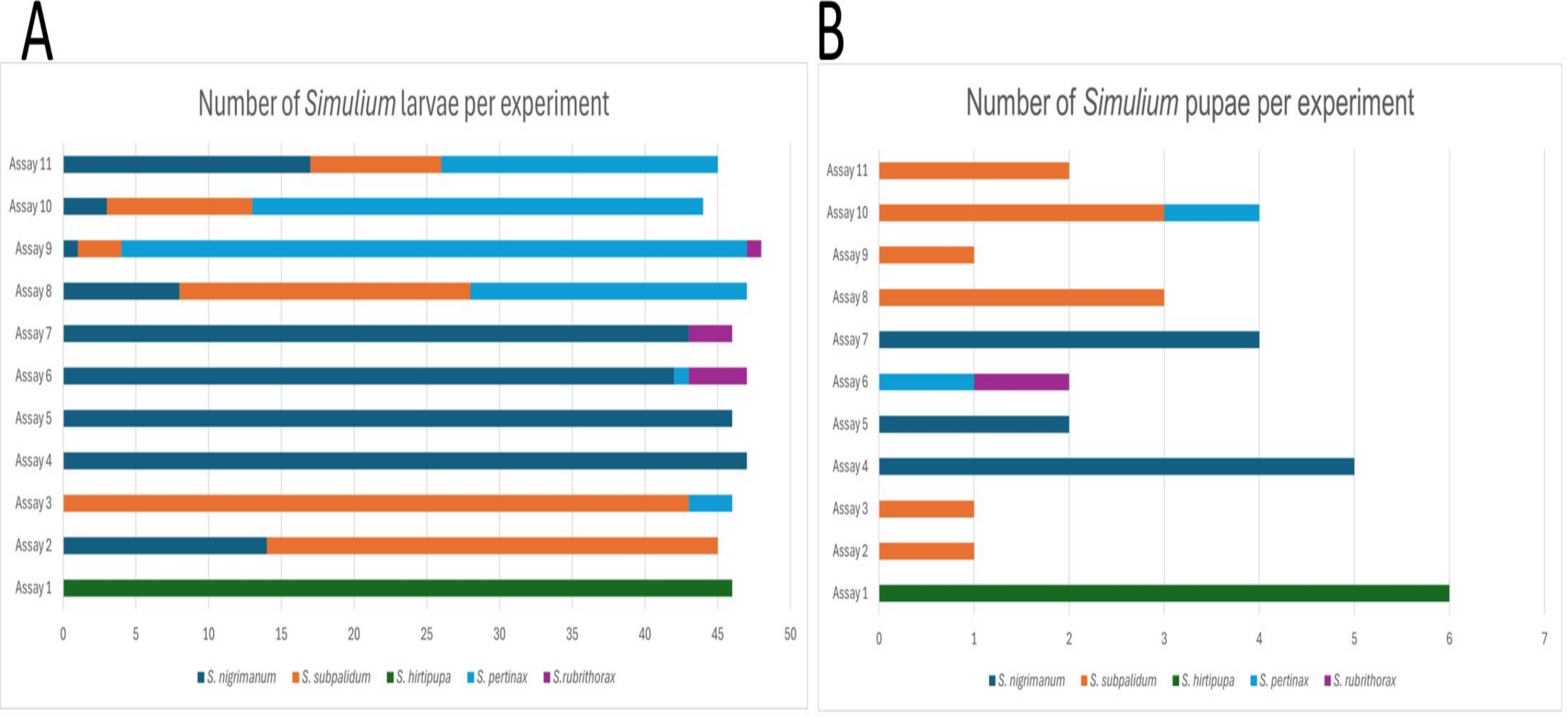
Species composition and pupation success of *Simulium* spp. maintained under laboratory rearing conditions. (A) Relative abundance and species composition of *Simulium* larvae recorded across 11 independent rearing assays conducted in circular-flow aquaria. Each bar represents the total number of larvae per assay, with colors indicating species identity. The data illustrate inter-assay variation in species representation and initial larval community structure. (B) Number of pupae obtained from each assay, showing successful completion of larval development under laboratory conditions. Species-specific differences in pupation output are evident, reflecting variation in developmental performance and adaptation to the artificial rearing system.

**Figure 5:**
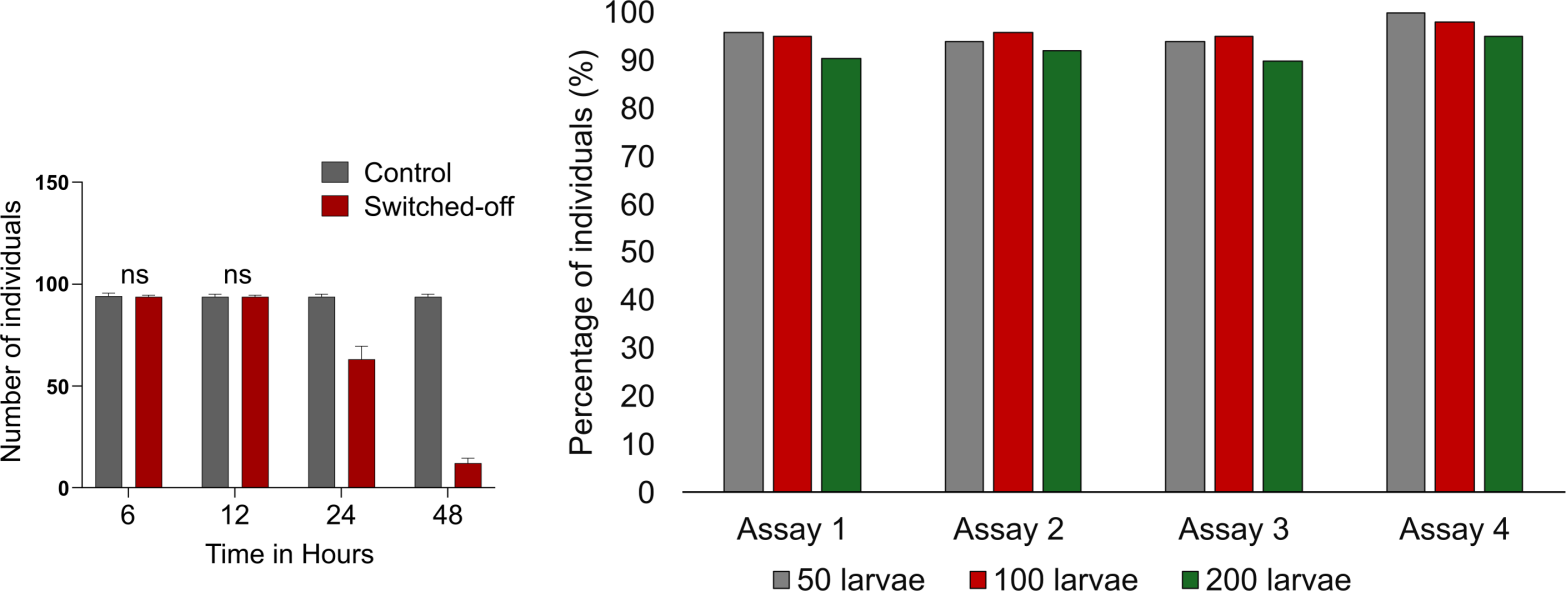
Effects of water circulation and larval density on survival of *Simulium* larvae under laboratory rearing conditions. (A) Survival dynamics of larvae maintained under continuous water circulation (control) compared with systems in which water flow was interrupted (switched-off). No significant differences were observed during the initial 6–12 h period (ns), whereas prolonged interruption resulted in a marked decline in larval survival after 24–48 h, demonstrating the dependence of *Simulium* larvae on sustained flow conditions. (B) Survival percentages obtained in density-dependent assays performed with 50, 100, and 200 larvae per aquarium across four independent experiments. Comparable survival rates among densities indicate that the circular-flow rearing system maintains stable environmental conditions and supports elevated larval loads without substantial density-dependent mortality.

**Figure 6:**
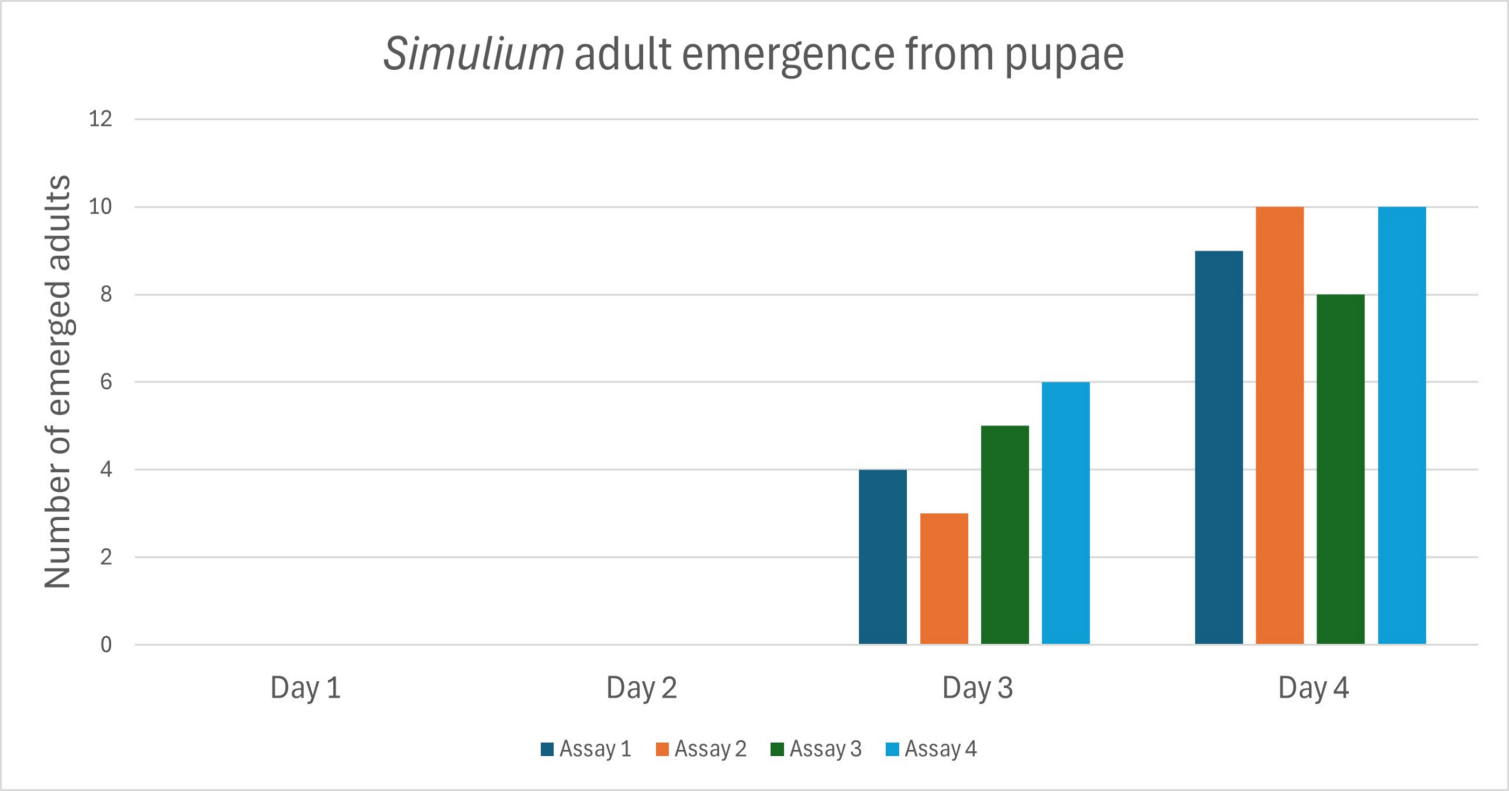
Temporal dynamics of adult emergence from laboratory-reared *Simulium* pupae. Daily emergence of adult individuals recorded over a four-day period following pupation under controlled laboratory conditions. Adult emergence initiated on Day 3 and peaked on Day 4, indicating synchronized developmental progression and high pupal viability within the circular-flow rearing system. The clustered emergence pattern suggests stable environmental conditions supporting successful completion of metamorphosis from pupae to adults.

The laboratory system efficiently supported all aquatic developmental stages of *Simulium* species, from egg incubation to adult emergence. Environmental parameters remained stable throughout the experiments, with temperature ranging from 20–25 °C, pH maintained near neutrality, and ammonia and nitrite concentrations consistently below 0.25 ppm. High survival rates were observed across developmental stages, allowing the assessment of system performance under different experimental conditions (Figs. 4, 5 and 6).

### Egg transport and hatching efficiency

Egg masses collected in the São Pedro River (Fig. 1) were successfully transported and incubated using an aerated portable system followed by fixed aeration in the bioterium until larval hatching (Fig. 2). Egg viability was preserved during transport, with no visible deterioration or fungal contamination observed. Across four independent hatching assays, the average larval hatching success reached 74% (Table 1). Eggs incubated under controlled aeration and stable physicochemical conditions hatched consistently within the incubation period, and newly emerged larvae displayed normal attachment and motility behavior. These results indicate that the egg transport and incubation setup provided suitable conditions for embryonic development.

**Table 1:** Egg hatching rates of *Simulium* spp. in four independent assays. Percentage of eggs hatched in each of four experimental trials conducted under controlled laboratory conditions. The average hatching success across all assays was 74%, indicating the effectiveness of the aeration and incubation protocol in the simulated lotic environment.

	Assay 1	Assay 2	Assay 3	Assay 4
Hatching (%)	87	70	71	67

### Species composition and larval development in artificial aquaria

Morphological identification revealed that multiple *Simulium* species developed concurrently within the laboratory rearing system (Fig. S1). Five taxa were consistently identified across the experiments: *Simulium nigrimanum* Macquart, 1838, *S. subpallidum* Lutz, 1910, *S. hirtipupa* Lutz, 1910, *S. pertinax* Kollar, 1832, and *S. rubritorax* Lutz, 1909. Species composition varied among assays, reflecting the natural heterogeneity of egg masses collected in the field (Fig. 4A). Larval survival remained high across experiments, with dominant species representing a substantial proportion of individuals reaching later developmental stages. The maintenance of multispecies assemblages indicates that the rearing system supported the development of naturally co-occurring taxa without strong exclusion effects under the conditions tested.

### Pupal development and species-specific outcomes

The number of individuals reaching the pupal stage varied among species and experiments (Fig. 4B). *Simulium nigrimanum* consistently exhibited the highest pupation success, while *S. subpallidum* showed intermediate but variable performance across assays. The remaining species were present at lower frequencies, although *S. hirtipupa* displayed a marked peak in pupal abundance in one experiment. Despite interspecific variation, all identified species successfully completed larval development and reached pupation under laboratory conditions, demonstrating that the system supports the full aquatic developmental cycle of multiple *Simulium* species.

### Dependence of larval survival on continuous water flow

The interruption of water circulation had a pronounced effect on larval survival (Fig. 5A). In control aquaria with continuous flow and aeration, survival rates remained above 90% throughout the 48-h observation period. In contrast, larvae subjected to flow cessation exhibited a time-dependent decline in survival. After 24 h without circulation, survival decreased to approximately 65%, and after 48 h, survival dropped sharply to around 10%. Increased variability among replicates was observed at later time points, indicating heterogeneous tolerance to stagnant conditions. These results demonstrate the critical importance of continuous water flow for maintaining larval viability in laboratory aquaria.

### System robustness across larval densities

Larval density assays showed that the rearing system supported development across a range of initial densities (50, 100, and 200 larvae per aquarium; Fig. 5B). Mean larval survival across all assays was 92%, and the average time from hatching to pupation was 13 days (Table 2). Although survival declined slightly at the highest density (91.5% at 200 larvae), larvae developed successfully under all tested conditions. These findings indicate that the system is robust to moderate increases in larval density while maintaining high developmental success.

**Table 2:** Larval survival rates (%) across 11 *Simulium* spp. rearing assays. Survival rates of Simulium larvae in 11 independent rearing experiments using the dual- container circular flow system. The average larval survival rate was 92%, highlighting the robustness and stability of the rearing setup under standardized environmental conditions (25 °C, pH 7, ammonia/nitrite < 0.25 ppm).

	Assay 1	Assay 2	Assay 3	Assay 4	Assay 5	Assay 6	Assay 7	Assay 8	Assay 9	Assay 10	Assay 11
Total	92	90	92	94	92	94	92	94	96	88	90

### Adult emergence from laboratory-produced pupae

Pupal viability assays revealed high adult emergence rates under controlled laboratory conditions (Fig. 6). Across four independent assays, the mean emergence rate reached 92.5% over a four-day monitoring period. Adult emergence followed a clear temporal pattern, with low numbers during the initial days and peak emergence occurring toward the end of the assay. The synchronized emergence of adults indicates that pupae produced in the laboratory retained normal developmental timing and viability. These results confirm the capacity of the rearing system to generate viable adult *Simulium* individuals from laboratory-reared larvae.

### Summary of developmental performance

Taken together, the results demonstrate that the proposed laboratory system reliably supports egg hatching, larval development, pupation, and adult emergence of multiple *Simulium* species (Fig. S1). High survival rates were maintained across developmental stages, and the system remained functional under varying larval densities and experimental manipulations. This performance establishes the system as a robust platform for experimental studies involving simuliid development under controlled conditions.

## Discussion

### A reproducible framework for laboratory rearing of *Simulium* species

The main contribution of this study is the establishment and validation of a reproducible laboratory system capable of supporting the complete aquatic development of *Simulium* species, from egg hatching to adult emergence. Despite the recognized medical, ecological, and economic importance of black flies, experimental studies on their development under controlled laboratory conditions remain comparatively scarce, largely due to the technical challenges associated with maintaining lotic environments outside natural streams (Brenner & Cupp, 1980; Edman & Simmons, 1985; Gray & Noblet, 2014). Our results demonstrate that these constraints can be effectively addressed using a relatively simple system combining continuous water circulation, aeration, and basic physicochemical monitoring. Importantly, the present study does not aim to establish a long-term laboratory colony or to maximize productivity. Instead, it focuses on identifying the minimal conditions required to sustain development and viability across life stages, which represents a prerequisite for experimental work involving simuliid larvae and pupae (Adler & McCreadie, 2019).

### Egg hatching as a critical developmental bottleneck

Egg hatching is widely recognized as a critical bottleneck in the laboratory rearing of aquatic insects, particularly taxa adapted to highly oxygenated lotic habitats (Schneider et al., 2003). In the present study, mean hatching rates exceeded 70% across independent assays, indicating that the incubation protocol was both effective and reproducible. These values are comparable to those reported in previous laboratory studies of Simuliidae and other lotic Diptera (Edman & Simmons, 1985; Gray & Noblet, 2014). The use of aerated incubation containers and water conditioned from the collection site likely contributed to the observed hatching success. Moreover, the separation of hatching-rate assays from larval rearing experiments avoided methodological overlap that could compromise interpretation, an issue previously noted in early rearing attempts for black flies (Brenner & Cupp, 1980).

### Larval survival and the role of hydrodynamic conditions

Larval survival remained high under continuous water circulation, with mean survival rates exceeding 90%. The flow-interruption experiments provided a direct experimental demonstration of the dependence of simuliid larvae on sustained water movement. Survival declined markedly after 24 h without circulation and dropped sharply after 48 h, underscoring that even short-term disruption of hydrodynamic conditions is incompatible with larval viability. These results are consistent with the obligate filter-feeding ecology of simuliid larvae, which rely on flowing water for oxygen delivery, food transport, and waste removal (Brenner & Cupp, 1980; Adler & McCreadie, 2019). Similar dependencies have been documented in field-based studies examining larval distribution and habitat use in tropical streams (Hamada, Adler & McCreadie, 2018).

### Density tolerance and system robustness

The rearing system supported larval development across a range of initial densities, with only a modest decline in survival at the highest density tested. Density-dependent effects on survival and development have been previously reported for simuliid larvae in both laboratory and natural settings (Srisuka et al., 2015; Hamada, Pepinelli & Gil-Azevedo, 2022), and the patterns observed here are consistent with these reports. The objective of the density assays was not to define optimal density thresholds, but to evaluate the robustness of the system under experimentally relevant conditions. Further optimization of density-dependent performance would require dedicated studies beyond the scope of the present work.

### Multispecies development under laboratory conditions

A notable outcome of this study was the concurrent development of multiple *Simulium* species within the same laboratory rearing system. Species composition varied among assays, reflecting the natural heterogeneity of egg masses collected in the field. Field surveys have shown that simuliid species frequently coexist at oviposition sites and share larval microhabitats in natural streams (Adler & Crosskey, 2020; Adler, Cheke & Post, 2010; Hamada, Pepinelli & Gil-Azevedo, 2022). Despite interspecific variation in larval survival and pupation rates, all identified species successfully completed larval development and reached the pupal stage. Importantly, no inferences are made regarding interspecific interactions, competitive hierarchies, or ecological equivalence, as these processes were not explicitly tested in the present study.

### Pupal viability and adult emergence

High pupal viability and synchronized adult emergence further support the suitability of the rearing system. Emergence rates above 90% are comparable to those reported in established laboratory protocols for Simuliidae (Gray & Noblet, 2014) and indicate that pupae produced under laboratory conditions retain normal developmental competence. The temporal pattern of adult emergence observed across assays suggests coordinated pupal development once larvae reached the pupal stage. Although adult mating, oviposition, and colony establishment were not evaluated, the reliable production of viable adults represents a critical prerequisite for future experimental applications requiring adult material.

## Conclusions

In summary, this study presents a reproducible and efficient method for rearing *Simulium* species under laboratory conditions, supporting high survival across developmental stages and the concurrent development of multiple species. By clearly defining the environmental requirements for successful rearing, this work provides a solid methodological foundation for future experimental studies involving black flies.

## Supplemental Information

10.7717/peerj.21193/supp-1 Supplemental Information 1 Raw Data

10.7717/peerj.21193/supp-2 Supplemental Information 2 Pupal morphology of five *Simulium* species reared under laboratory conditions Lateral view of pupae from distinct *Simulium* species: (A) *Simulium hirtipupa*; (B) *Simulium nigrimanum*; (C) *Simulium rubrithorax*; (D) *Simulium pertinax*; and (E) *Simulium subpallidum*. Interspecific variation is evident in the number, length, thickness, and spatial arrangement of the cephalic respiratory filaments, as well as in abdominal pigmentation and overall body morphology. These characters are consistent with diagnostic traits used in Simuliidae taxonomy. Scale bars = two mm.

## Additional Information and Declarations

### Data Availability

The following information was supplied regarding data availability: The raw data is available in the Supplemental Files.
