# Supplementary figures and images for: A laboratory system for rearing Simuliidae (Diptera) under simulated lotic environments
Source: PeerJ. 2026 Jun 8;14:e21193. doi: 10.7717/peerj.21193 (PMC13256054; doi:10.7717/peerj.21193)

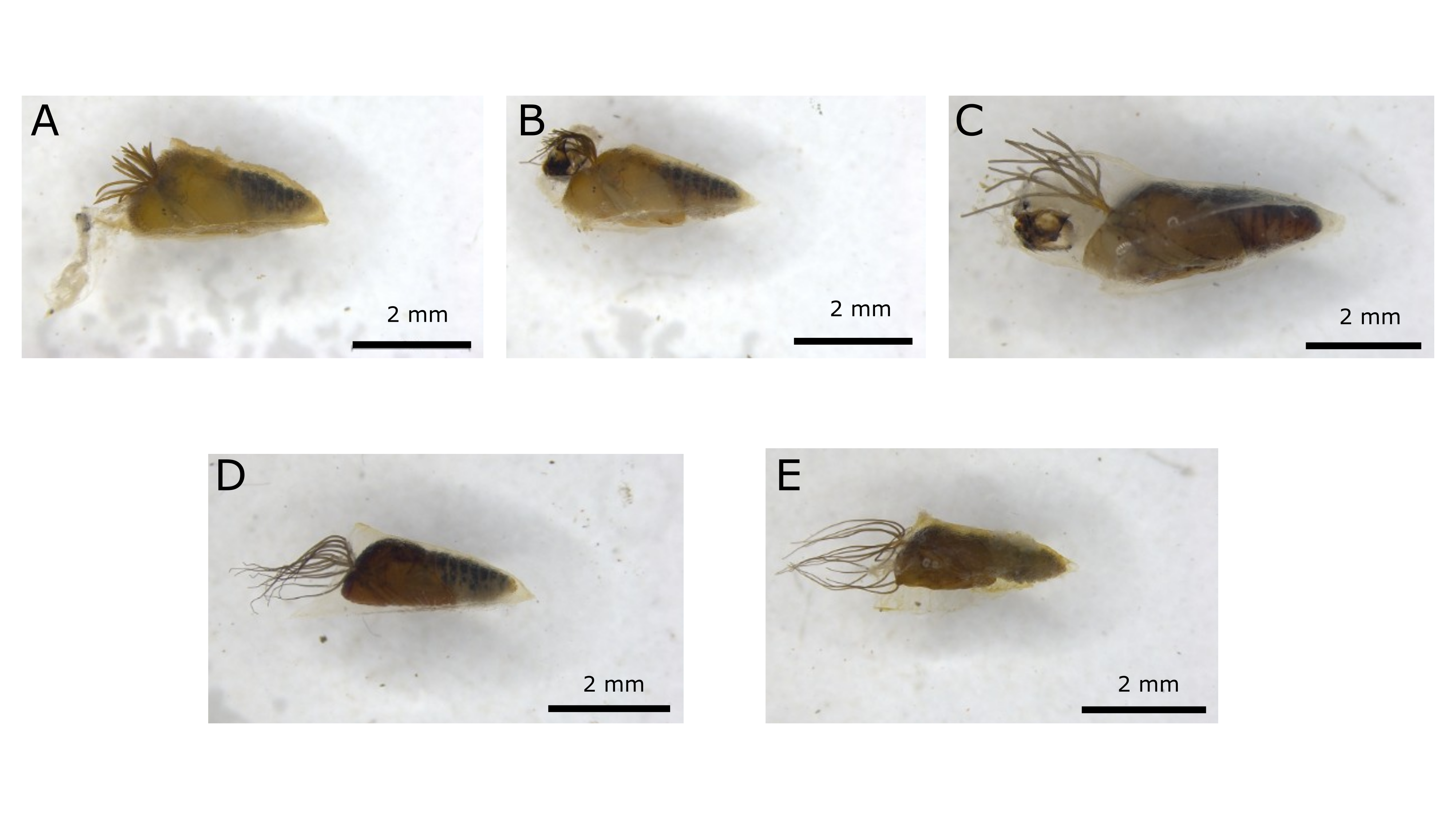

Supplement: Supplemental Information 2 — Lateral view of pupae from distinct Simulium species: (A) Simulium hirtipupa; (B) Simulium nigrimanum; (C) Simulium rubrithorax; (D) Simulium pertinax; and (E) Simulium subpallidum. Interspecific variation is evident in the number, length, thickness, and spatial arrangement of the cephalic respiratory filaments, as well as in abdominal pigmentation and overall body morphology. These characters are consistent with diagnostic traits used in Simuliidae taxonomy. Scale bars = two mm. [file peerj-14-21193-s002.png]
